# Silibinin Ameliorates Formaldehyde-Induced Cognitive Impairment by Inhibiting Oxidative Stress

**DOI:** 10.1155/2022/5981353

**Published:** 2022-06-16

**Authors:** Pengsheng Wei, Xue Li, Shuai Wang, Yanxin Dong, Haoran Yin, Zikun Gu, Xiaoting Na, Xi Wei, Jiayu Yuan, Jiahui Cao, Haotian Gao, Yebo Su, Yong Xu Chen, Ge Jin

**Affiliations:** ^1^Basic Medical School, Shenyang Medical College, China; ^2^School of Pharmacy, Shenyang Medical College, China; ^3^Key Laboratory of Behavioral and Cognitive Neuroscience of Liaoning Province, Shenyang Medical College, China

## Abstract

Silibinin is a flavonoid extracted from the medicinal plant Silybum marianum (milk thistle), traditionally used to treat liver disease. Recent studies have shown that the antioxidative stress and anti-inflammatory effects of milk thistle are used in the treatment of neurological diseases. Silibinin has antioxidative stress and antiapoptotic effects and reduces cognitive impairment in models of Alzheimer's disease (AD). However, the underlying mechanism of silibinin related to improvement of cognition remains poorly understood. In this study, we used the model of lateral ventricle injection of formaldehyde to examine the related mechanism of silibinin in improving cognitive impairment disorders. Oral administration of silibinin for three weeks significantly attenuated the cognitive deficits of formaldehyde-induced mice in a *Y*-maze test and Morris water maze test. *Y*-maze results show that silibinin increases the rate of spontaneous response alternation in FA-induced mice. Silibinin increases the target quadrant spending time and decreases escape latency in the Morris water maze test. We examined the effect of silibinin on the NRF2 signaling pathway, and silibinin promoted the nuclear transfer of NRF2 and increased the expression of HO-1 but did not significantly increase the protein expression of NRF2 in the hippocampus. Well, silibinin reduces the content of DHE and decreases the levels of apoptosis of mature neuron cells. We investigated the effect of silibinin on the content of formaldehyde degrading enzymes; biochemical analyses revealed that silibinin increased GSH and ALDH2 in formaldehyde-induced mice. In addition, as one of the pathological changes of AD, TAU protein is also hyperphosphorylated in FA model mice. Silibinin inhibits the expression of GSK-3*β* in model mice, thereby reducing the phosphorylation of TAU proteins ser396 and ser404 mediated by GSK3*β*. Based on our findings, we verified that the mechanism of silibinin improving cognitive impairment may be antioxidative stress, and silibinin is one of the potentially promising drugs to prevent formaldehyde-induced cognitive impairment.

## 1. Introduction

Formaldehyde (FA), a member of the aldehyde family, is an important chemical material, and it has been widely used in building materials, household products, chemical synthesis, and medicinal applications [1, 2]. However, the current misuse of formaldehyde has exceeded the threshold, especially in developing countries. In the food industry, formaldehyde is used as an additive in aquatic products, agricultural and sideline products, and alcohol. Formaldehyde is harmful to human health, and it has attracted increasing attention as one of the most important pollutants in the last decades [3, 4]. It has been reported that FA has toxic effects on the central nervous system and has been classified as having probable neurotoxicity [5]. Long-term exposure to FA may cause neurotoxicity and result in neurodegenerative disorders [6]. FA may cause various morphological changes in rat brains and result in behavior and memory disorders [7, 8]. It is an urgent task to find the mechanisms of the noxious effects of FA and an effective protective agent against neurotoxicity induced by formaldehyde. Oxidative stress has been shown to be a major contributor to the progression of neurodegenerative disease [9]. Oxidative damage caused by free radicals is one of the important molecular mechanisms of toxic effects of chemical poisons. The accumulation of free radicals in the body can cause oxidative damage to biological macromolecules, such as proteins and lipid membrane. For the last two decades, oxidative stress has been recognized and discussed as a major factor in AD pathogenesis, with several review articles highlighting the critical role that reactive oxygen species (ROS) play in AD pathology [10, 11]. Silibinin is a flavonoid derived from the medicinal plant Silybum marianum (milk thistle) and has traditionally been used to treat liver diseases [12]. In addition, silibinin can act as an antioxidant to combat oxidative stress-related neuropathy [13]. The results of our previous studies have shown that silibinin reduces A*β* deposition and has anti-inflammatory and antiapoptotic effects that improve cognitive impairment in AD model mice [14, 15]. In this work, we used intracerebroventricular injection of formaldehyde in C57 mice as an animal model for three weeks of continuous administration to explore the effect of silibinin on improving spatial learning and memory impairment and its related mechanisms.

## 2. Materials and Methods

### 2.1. Chemicals

Silibinin was purchased from Green Biological Development Co., Ltd. (Panjin, China), and formaldehyde (FA) solution (AR) was purchased from Xilong Chemical (Shantou City, China).

### 2.2. Animals and Groups

**Figure 1:**
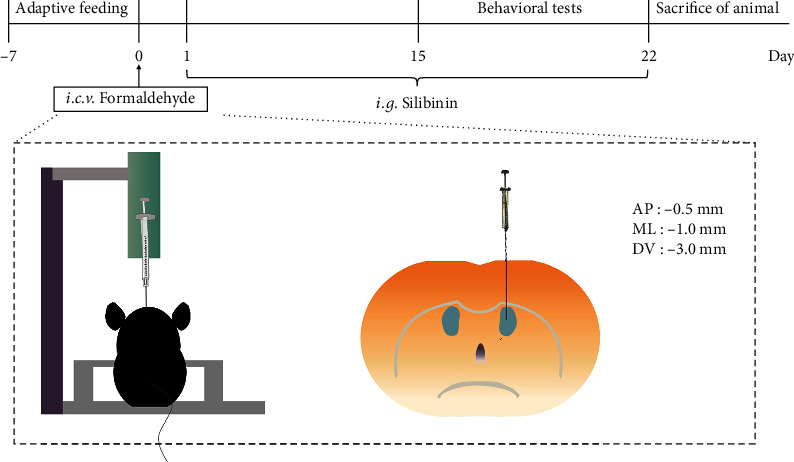
Experimental flow chart and intracerebroventricular injection diagram.

Adult C57 mice were purchased from Liaoning Changsheng Experimental Animal Center (Shenyang, China) and housed in standard environmental conditions (12 h light-dark cycle, 50%–60% humidity, and 22–25°C). The mice had free access to food and water. Mice acclimatized for a week before the start of the experiment. Mice were randomly divided into five groups: sham group, FA 2 mM group, FA 2 mM + silibinin 100 mg/kg group, FA2mM + silibinin 200 mg/kg group, and the memantine 2.6 mg/kg group. The dose of silibinin in this study was based on our previous study [14, 15]. There were ten mice per group with five males and five females each. The sham group received an intracerebroventricular injection of normal saline. The FA group received a 2 ulintracerebroventricular injection of FA which was performed according to our previous article with the following coordinates from bregma: AP: −0.5 mm; ML: −1.0 mm; and DV: −3 mm [16]. The silibinin groups were given silibinin on the second day after the intracerebroventricular injection. The memantine group was treated with 2.6 mg/kg memantine. The sham group and the model groups were given the same amount of solvent. The method of administration was gavage for three consecutive weeks (Figure 1). All animal studies were performed in strict accordance with the Guide for the Care and Use of Laboratory Animals (P.R. China legislation on the use and care of laboratory animals: Permit Number: SYPU-IACUC-C2015-0831-203). Animal experiments were performed at Shenyang Pharmaceutical University.

## 3. Behavioral Tests

### 3.1. *Y*-Maze Test

The *Y*-maze test was performed after 14 days of intracerebroventricular injection as described in our previous report [14]. The maze was composed of three arms (A, B, and C). Each arm was 40 cm long, 12 cm tall, and 10 cm wide. Each mouse was first placed at the end of the A arm and allowed to move freely to the other arms for five minutes. The number of mice entering each arm was recorded. Alternation was defined as the successful consecutive entry of the mouse into each of the three arms. The alternation behavior (%) was calculated using the equation (number of successful alternations/(total number of arms entries–2) × 100).

### 3.2. Morris Water Maze (MWM) Test

The MWM test was performed after 15 days of intracerebroventricular injection. The apparatus was a white plastic circular pool (100 cm in diameter and 50 cm deep) filled with water (23 ± 1°C and 40 cm deep). A hidden circular platform (10 cm in diameter, placed 1 cm below the water surface) was in one of the four equal quadrants. Mice were trained twice a day for five consecutive days with an intertrial interval of 6 h. Animals were placed in the water facing the wall individually and were allowed 60 s to find the hidden platform. If the mouse failed to find the platform, the experimenter placed the mouse on the platform for 10 s. The platform was removed on the sixth day; mice were individually administered a probe test as in a previous experiment [14]. A computer system (equipped with a video camera) automatically captured the escape latency (time spent in the target quadrant) and swimming distance. After the MWM test, mice were decapitated under isoflurane anesthesia, and the brain tissue was dissected and stored at -80°C.

## 4. Immunohistochemistry and Immunofluorescence

The mice were perfused at the indicated time points after the behavioral experiment with cold normal saline and fixed with 4% paraformaldehyde. The brains were embedded in paraffin and then cut into 10 *μ*m sections. The sections at specific levels were selected for immunohistochemical and immunofluorescence stains. After being blocked with 5% goat serum for 1 h, sections were stained with anti-NeuN (1 : 1000, Abcam), and TUNEL staining was performed using the One Step TUNEL Apoptosis Assay Kit (Beyotime, China), following the kit instructions to detect red fluorescence in NeuN-labeled cells. DHE staining used dihydroethidium staining solution (DHE, Beyotime, China) incubated in the dark for 30 min. After incubation, the sections were washed 3 times with PBS, and the red fluorescence was observed. Nrf2 (1 : 500; ABclonal) and nuclei were stained with 4′6-diamidin-2-phenylindol (Antifade Mounting Medium with DAPI, Beyotime, China) for Nrf2 immunofluorescence. GSK3*β* (1 : 1000, Santa Cruz) antibodies were probed overnight at 4°C and incubated with secondary antibodies at 37°C for 1 h. NeuN-positive cell numbers,Nrf2 fluorescence intensity, and GSK3*β* were counted with ImageJ.

## 5. Determination of the Presence of Formaldehyde Metabolizing Enzymes

The production of enzymes was detected using an ELISA assay kit (Wuhan Meimian Institute of Biotechnology, China) according to the manufacturer's instructions. Briefly, when the drug intervention was completed, the mice were killed, and the prefrontal cortices and hippocampi were dissected. The samples were homogenized in 2 mM PBS with a high throughput homogenizer. The homogenates were centrifuged at 3,500 g for 20 min at 4°C. The supernatant was used for the following analyses: the concentration of alcohol dehydrogenase 3 (ADH3), aldehyde dehydrogenase 2 (ALDH2), and glutathione (GSH).

## 6. Western Blotting Analysis

Brain tissues were prepared in the same manner as the samples for cytokine assays using an ELISA. Samples were subjected to PAGE gel electrophoresis (PAGE; 10% gel in MOPS buffer). Proteins were transferred to a polyvinylidene difluoride membrane (Bio-Rad, CA, USA) by electroelution and then separately incubated with rabbit anti-*β*-actin (1 : 1,000; ABclonal, China), rabbit Ho-1 antibodies (1 : 1,000; ABclonal, China), rabbit Nrf2 antibodies (1 : 600; ABclonal, China), and rabbit p-GSK-3*β*, p-Tau ser396, and ser404 antibodies (1 : 1,000, Santa Cruz). Subsequently, protein bands were detected using a chemiluminescence kit (Ncm ECL Ultra kit; New Cell & Molecular Biotech, China).

## 7. Results

**Figure 2:**
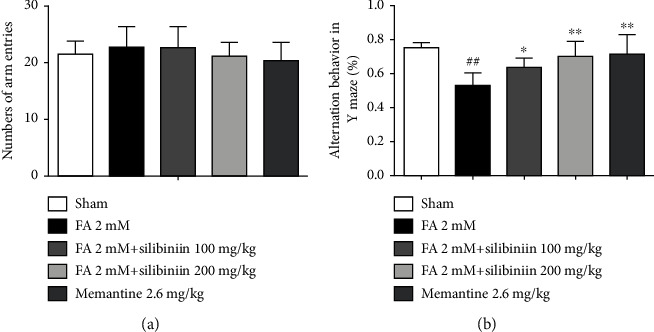
Silibinin significantly increased spontaneous alternation behaviors informaldehyde-induced cognitive impairment mice. (a) The total number of arm entries in the *Y*-maze test. (b) Alternation (%) in the *Y*-maze test. The data are presented as the mean ± SEM and were analyzed with one-way ANOVA, *N* = 10 animals per group, ##*p* < 0.01 compared with the sham group; ^∗^*p* < 0.05 and ^∗∗^*p* < 0.01 compared with the FA group.

Silibinin increased spontaneous alternation behavior in the *Y*-maze of formaldehyde-induced cognitive impairment model mice (Figure 2).

**Figure 3:**
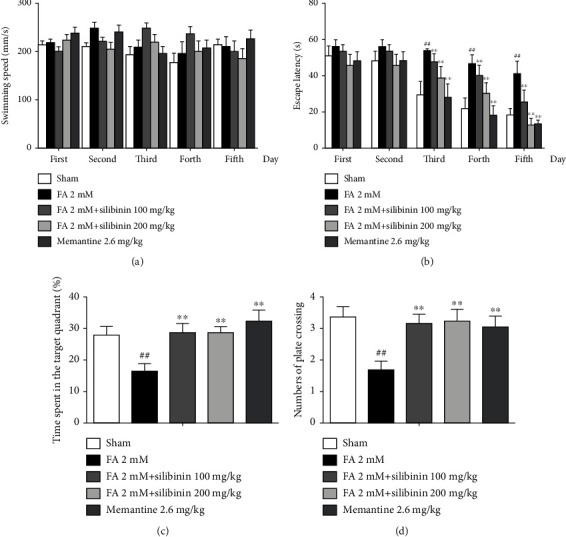
Silibinin significantly increased spatial memory ability in formaldehyde-induced cognitive impairment mice. (a) Swimming speed in the training period. (b) Escape latency in the training period. (c) Mean time spent in the target quadrant in the probe trial. (d) Mean number of platform crossings during the probe trial. The data are presented as the mean ± SEM and were analyzed with one-way ANOVA, *N* = 10 animals per group, ##*p* < 0.01 compared with the sham group; ^∗∗^*p* < 0.01 compared with the FA group.

Silibinin increased spatial memory ability in the Morris water maze test of formaldehyde-induced cognitive impairment model mice (Figure 3).

**Figure 4:**
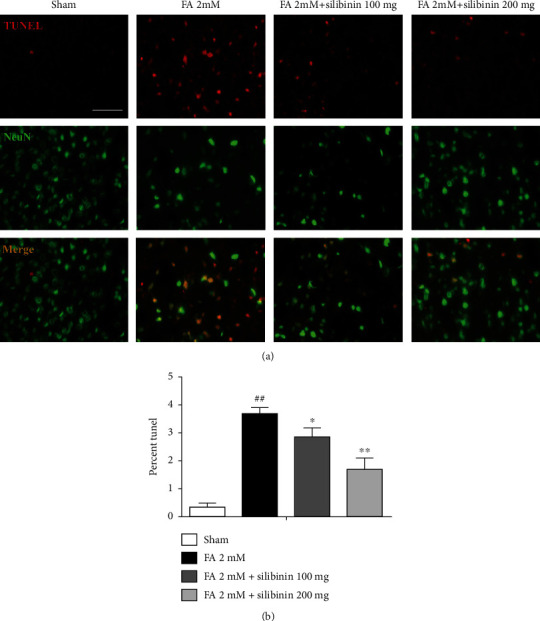
The levels of apoptosis, as assessed by TUNEL staining (red), were significantly higher in NeuN+ (green) cells of the FA group when compared to the sham group. Levels in the silibinin group were significantly decreased compared to the FA group. (a) The levels of apoptosis. (b) Quantification of the frequency of TUNEL events in NeuN+ cells. The data are presented as the mean ± SEM and were analyzed with one-way ANOVA, *N* = 5 animals per group, ##*p* < 0.01 compared with the sham group; ^∗^*p* < 0.05, ^∗∗^*p* < 0.01 compared with the FA group.

Silibinin decreased the level of apoptosis of mature neuron cells (Figure 4).

**Figure 5:**
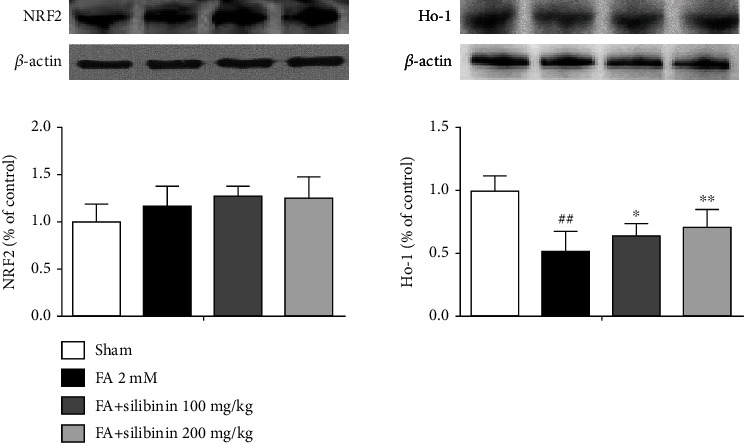
Silibinin increased the expression of Ho-1 protein in the hippocampus. The data are presented as the mean ± SEM and were analyzed with one-way ANOVA, *N* = 4 mice per group, ##*p* < 0.01 compared with the sham group; ^∗^*p* < 0.05, ^∗∗^*p* < 0.01, compared with the FA group.

Silibinin increased the expression of HO-1 protein in formaldehyde-induced cognitive impairment model mice (Figure 5).

**Figure 6:**
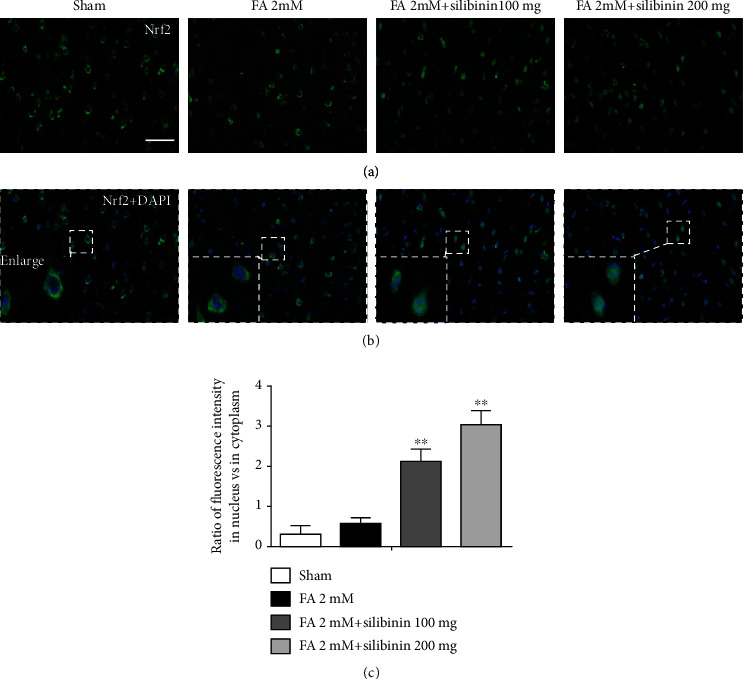
Silibinin promoted Nrf2 translocation from the cytoplasm to the nucleus in FA treated mice. Nrf2 was marked using specific antibody (green) immunofluorescence. Nuclei were labeled using DAPI (blue). Typical photographs of merging and enlarging are shown (a, b). Five animals were used for each group, and three photographs were analyzed for each animal. Scale bars represent 50 *μ*m, and the magnification of the merge was ×400. Silibinin promoted Nrf2 translocation from the cytoplasm to the nucleus, increasing the proportion of Nrf2 localization in the nucleus. Quantification of the Nrf2 fluorescence intensity in the nucleus and cytoplasm (c). The data are presented as the mean ± SEM and were analyzed with one-way ANOVA, ^∗∗^*p* < 0.01 compared with the FA group.

Silibinin promoted Nrf2 translocation from the cytoplasm to the nucleus (Figure 6).

**Figure 7:**
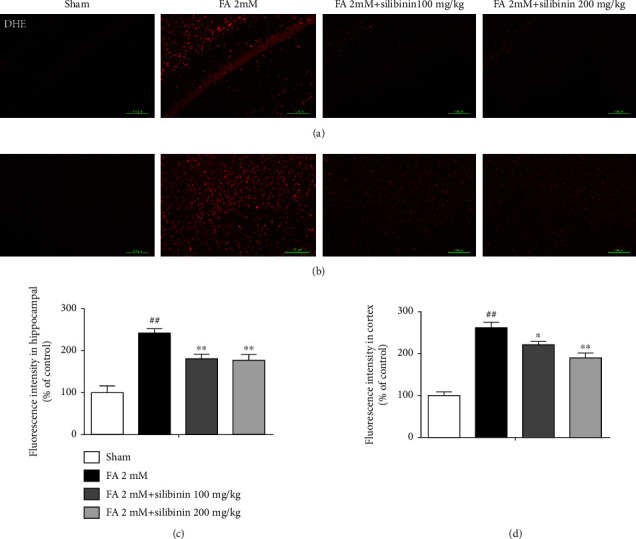
Silibinin decreased reactive oxygen species (ROS) content in FA-induced mouse brains. ROS were labeled using DHE (red). The higher red fluorescence intensity per field represents greater ROS content. Fluorescence intensities of DHE in the hippocampal CA1 (a) and cortex (b) are shown. Scale bars represent 100 *μ*m. Quantifications of the fluorescence intensity of DHE are shown in (c) and (d). The data are presented as the mean ± SEM and were analyzed with one-way ANOVA, *N* = 5 animals per group, ##*p* < 0.01 compared with the sham group; ^∗^*p* < 0.05 and ^∗∗^*p* < 0.01 compared with the FA group.

Silibinin reduces the content of ROS in formaldehyde-induced cognitive impairment model mice (Figure 7).

**Figure 8:**
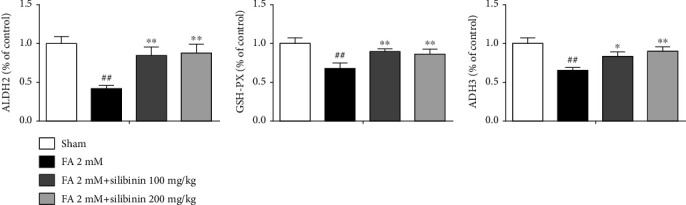
Silibinin increased the expression of ALDH2, GSH, and ADH3 proteins in the cortex of formaldehyde-induced cognitive impairment model mice. The data are presented as the mean ± SEM and were analyzed with one-way ANOVA, *N* = 4 animals per group, ##*p* < 0.01 compared with the sham group; ^∗^*p* < 0.05 and ^∗∗^*p* < 0.01 compared with the FA group.

Silibinin increased the expression of ALDH2, GSH, and ADH3 proteins in the cortex of formaldehyde-induced cognitive impairment model mice (Figure 8).

**Figure 9:**
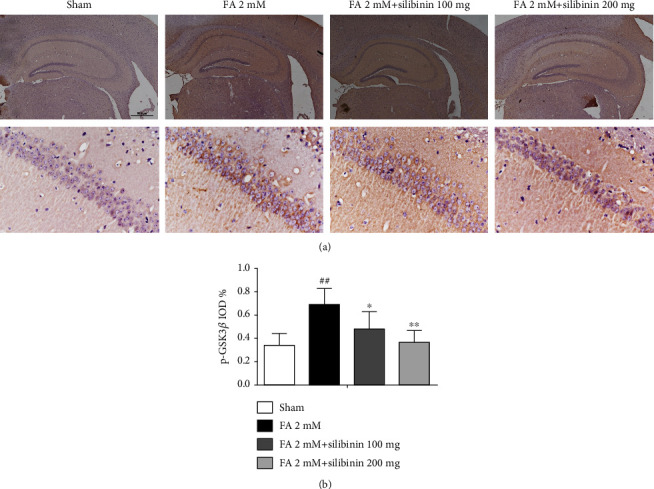
Silibinin reduced the expression of phosphorylated GSK3*β* (Y216). (a) The expression of phosphorylated GSK3*β* in the cortex and hippocampus. (b) Quantification of the intensity of GSK3*β*. The data are presented as the mean ± SEM and were analyzed with one-way ANOVA, *N* = 5 animals per group, ##*p* < 0.01 compared with the sham group; ^∗^*p* < 0.05, ^∗∗^*p* < 0.01, compared with the FA group.

Silibinin reduced the expression of phosphorylated GSK3*β* (Y216) in formaldehyde-induced cognitive impairment mice (Figure 9).

**Figure 10:**
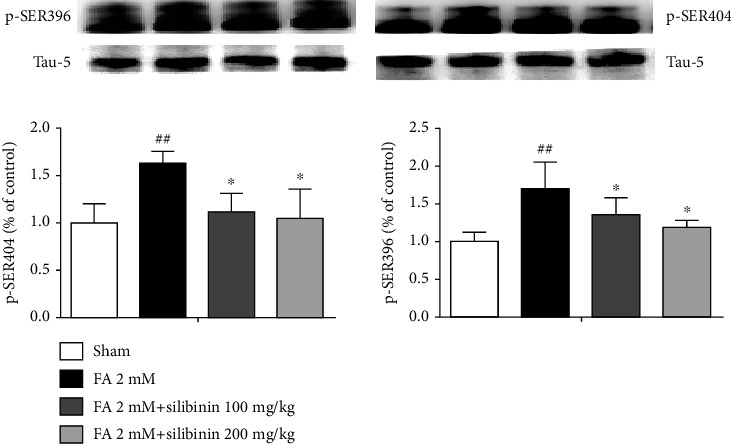
Silibinin inhibition of tau protein hyperphosphorylation of p-SER-396 and p-SER-404. The data are presented as the mean ± SEM and were analyzed with one-way ANOVA, *N* = 4 animals per group, ##*p* < 0.01 compared with the sham group; ^∗^*p* < 0.05, ^∗∗^*p* < 0.01, and ^∗∗∗^*p* < 0.001 compared with the FA group.

Silibinin inhibits the expression of tau protein hyperphosphorylation of p-SER-396 and p-SER-404 in the hippocampus of formaldehyde-induced cognitive impairment model mice (Figure 10).

## 8. Statistical Analysis

All data were presented as mean ± SEM. Differences between groups were determined by repeated one-way analysis of variance (ANOVA). Statistical differences were considered significant at *p* < 0.05.

## 9. Discussion

Accumulating evidence has demonstrated that exposure to formaldehyde causes neurotoxicity in animal models and humans [17, 18]. The neurotoxicity of formaldehyde includes impairment of the hippocampus [19] and changes in neurofilament proteins [20], hyperphosphorylation of tau proteins [21], and demyelization of hippocampal neurons. Formaldehyde is a small molecule substance (M.W. = 30) and is able to permeate the blood-brain barrier, which may explain why chronic exposure to formaldehyde induces wide neurotoxicity in the brain [22, 23]. However, the specific mechanisms and how to prevent cognitive function damage of formaldehyde need to be further explored. Our study showed that FA exposure could lead to the impairment of cognitive ability of C57 mice. Compared with the sham group, the formaldehyde group demonstrated working memory impairment and spatial learning and memory impairment in C57 mice. Silibinin increases the rate of spontaneous response alternation in *Y* maze and increases the target quadrant spending time and decreases escape latency in the Morris water maze test. Silibinin activates the Nrf2 pathway, a key antioxidative stress pathway that reduces oxidative stress. According to reports, excess intracellular ROS can cause damage to lipids, cell membranes, and organelles [24], which can lead to apoptosis. From our results, TUNEL-positive cells exhibited nuclear contraction and chromatin condensation. Silibinin treatment significantly reduced the number of TUNEL events in NeuN^+^ cells. The antiapoptotic effect of silibinin is consistent with our previous results [15].

Nuclear factor erythroid 2 p45-related factor 2 (Nrf2) is the key regulator of the cell against oxidative stress. As a transcription factor, Nrf2 can regulate the transcription of hundreds of genes, such as antioxidant stress genes and detoxification genes [25]. The Nrf2 protein has seven domains, of which the Neh1 domain has DNA binding ability, and the transcription of the antioxidative stress gene is initiated by combining with the antioxidant response element (ARE) [26]. Under physiological conditions, the Neh2 domain of Nrf2 binds to Kelch-like ECH-associated protein 1 (Keap1). Keap1 binds to E3 ubiquitin ligase and promotes ubiquitination and degradation of Nrf2 [27] and prevents the nuclear translocation of Nrf2 and the transcription of antioxidant stress genes such as heme oxygenase-1 (HO-1). HO-1 converts prooxidant heme to antioxidants and maintains the balance of the oxidative stress microenvironment. Interestingly, silibinin did not significantly increase the expression of NRF2 protein; so, we did a nuclear transfer experiment. The results showed that silibinin promoted Nrf2 translocation from the cytoplasm to the nucleus, reduced the content of DHE, and increased the protein level of HO-1, indicating that silibinin may increase the antioxidant capacity of cells by activating the Nrf2 pathway to promote the antioxidative stress effect.

Under physiological conditions, formaldehyde is mainly degraded by GSH-dependent ADH3. When the formaldehyde concentration in the body is abnormally increased, GSH-independent ALDH2 can also play an important degradation role. ALDH2 directly oxidizes formaldehyde to formic acid by using NAD+ as a coenzyme [28]. ADH3 and ALDH2, metabolizing enzymes of formaldehyde [29], are related to cognitive function. A decrease in ADH3 activity could lead to formaldehyde accumulation in brains with aging. The ALDH2 polymorphism, which can result in low activity of ALDH2 [30], is related to susceptibility to late-onset Alzheimer's disease. Our results showed that the expression of ADH3 and ALDH2 in the brain of the experimental animals in the silibinin group was increased when compared with FA group. Therefore, silibinin improves the cognitive impairment of the FA model mice, and this may be related to the increase in ADH3 and ALDH2 enzyme activities.

It has been reported that oxidative stress caused by formaldehyde is critical to its neurotoxicity [31]. Oxidative stress is defined as the imbalance between the production and elimination of ROS [32]. Glutathione is an important ROS scavenger; the active sulfhydryl group on cysteine is susceptible to oxidation by certain peroxides and free radicals [33]. The abnormally increased formaldehyde concentration consumes a large amount of GSH, which limits ROS scavenging and causes oxidative stress. The brain is the most susceptible organ to oxidative stress as it consumes more oxygen than any other organ and contains relatively low levels of antioxidant substances, while having high levels of phospholipids, which are vulnerable to oxidative damage [34]. Consequently, while both the brain and the nervous system are prone to oxidative stress, they are inadequately equipped with antioxidant defense systems to prevent ongoing oxidative damage [35]. Glutathione levels in the brain decrease during aging, and the decline of GSH in the brain is positively related to cognitive impairment [36, 37] in AD patients. In this study, we observed a silibinin significant increase of GSH in the FA group. This is probably one of the reasons formaldehyde causes oxidative stress in the brain.

Abnormal tau accumulation is positively correlated with neurodegeneration and memory deterioration [37], and the total tau level in cerebrospinal fluid has an inverse correlation with memory scores in AD patients [38, 39]. Axonal tau pathology in the hippocampus is critical for the clinical presentation of dementia and may constitute an anatomical substrate of clinically verifiable memory dysfunction [40, 41]. Human tau transgenic mice recapitulate the features of human tauopathies and cognitive deficits [42]. The mechanism of tau-mediated neurotoxicity occurs via two major mechanisms: toxic loss of function, in which physiological tau protein loses function causing microtubule destabilization [43], and toxic gain of function, in which highly phosphorylated tau display the toxic effects in neurons [44]. Furthermore, in vitro studies have demonstrated that soluble and prefibrillar tau oligomers possess far greater toxic characteristics than higher order tau. Tau is hyperphosphorylated at more than 38 different sites, which interferes with the affinity of tau microtubule binding [45] and causes neurofibrillary tangle formation [46]. Tau 396 and 404 are one of the common phosphorylation sites, and hyperphosphorylation of tau has also been observed in APP/PS1 transgenic mice [47]. There is evidence that formaldehyde causes tau hyperphosphorylation and aggregation in mouse or neuronal cell lines [48, 49]. GSK-3*β* is an evolutionarily conserved serine/threonine kinase and is one of the key kinases that phosphorylate tau [50]. Knocking down GSK3*β* at the gene level or using specific inhibitors can inhibit tau hyperphosphorylation [51, 52]. Phosphorylation of GSK3*β* at tyrosine 216 (Y216) can enhance the activity of phosphorylating downstream proteins such as tau. Tau becomes hyperphosphorylated not only in the cytoplasm but also in the nucleus of neuroblastoma (N2a) cells and mouse brain. Under formaldehyde induction, significant accumulation of glycogen synthase kinase-3*β* (GSK-3*β*) in N2a and mouse brain nuclei was observed, as well as increased phosphorylationatY216 [49]. In this study, formaldehyde hyperphosphorylated tau in the mouse brain, which is consistent with other research. Our results show that silibinin inhibits the expression of phosphorylated GSK3*β* (Y216), thereby inhibiting the hyperphosphorylation of tau 396 and 404. This suggests that silibinin may reduce tau hyperphosphorylation by inhibiting GSK3*β* (Y216), thereby improving cognitive dysfunction.

Generally, due to the low aqueous solubility of silibinin (less than 50 *μ*g/mL), silibinin bioavailability is poor [53]. Although silibinin is quickly absorbed, the absorption efficiency is low, and the absolute oral bioavailability of silibinin is about 0.95% [54, 55], while, on the other hand, clinical trials showed that patients were safe and well tolerated with high dose silibinin (0.42–2.1 g/day) [56]. In addition, there are brain uptake and accumulation of silibinin [57, 58] which may be responsible for various neuroprotective effects of silibinin [59]. In this study, we found that silibinin improved FA-induced cognitive impairments and inhibited FA-induced oxidative stress. Silibinin has been reported to ameliorate AD by multiple mechanisms such as inhibition of acetylcholinesterase activity, restrain of amyloid *β* peptide aggregation, regulation of gut microbiota, and suppression of oxidative stress [58, 60–63]. Importantly, accumulating evidence and our previous study suggest that silibinin ameliorated memory impairments in AD-like mice via suppression of oxidative stress [14, 15, 62, 63]. Similarly, in present study, we found that silibinin alleviated cognitive impairments probably via inhibiting oxidative stress in FA-treated mice. To explore the mechanism of silibinin inhibiting oxidative stress, we tested Nrf2, a key factor regulating oxidative stress homeostasis, and ADH3 and ALDH2, two key enzymes regulating formaldehyde metabolism. In the literature related, silibinin activated Nrf2 signaling to modulated oxidative stress [64, 65], and we showed similar results that silibinin increased Nrf2 nuclear translocation and transcription of downstream genes in FA-treated mice. Moreover, Nrf2 regulates the ALDH2 expression, and interestingly, ALDH2 can increase the Nrf2 expression, suggesting an NRF2-ALDH2 feedback loop [66–68]. There is, in addition, a cooperative function between ADH3 and NRF2 in regulating GSH level [69]. Therefore, it is interesting to further study the role of the complex relationship between ADH3, ALDH2, and Nrf2 in the regulation of oxidative stress by silibinin.

In summary, silibinin improves cognitive dysfunction caused by injection of formaldehyde into the lateral ventricle. These mechanisms may include silibinin increasing the expression of formaldehyde metabolic enzymes ADH3 and ALDH2, silibinin promoting nuclear transfer of NRF2 and reducing the oxidative stress response, and silibinin inhibiting the expression of phosphorylated GSK-3*β* (Y216) and thus inhibiting the hyperphosphorylation of tau's proteins.

## 10. Conclusions

**Figure 11:**
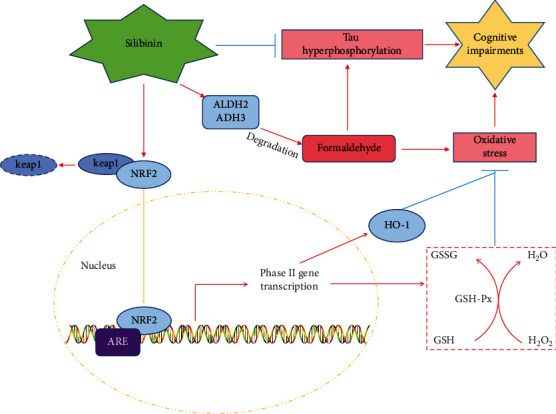
Molecular mechanisms of silibinin against FA-induced oxidative stress and cognitive impairments.

Our study indicated that silibinin rescues learning and memory impairment of FA induced mice. Silibinin increased the antioxidant capacity of cells by activating the Nrf2 pathway and promoting the antioxidative stress effect. Silibinin promoted Nrf2 translocation from the cytoplasm to the nucleus, reduced the content of DHE, increased the protein level of HO-1, and increased the expression of formaldehyde metabolic enzymes ADH3 and ALDH2. Silibinin inhibits the expression of phosphorylated GSK-3*β* and thus inhibits the hyperphosphorylation of tau's proteins. These experimental data lay a foundation for understanding the pathogenesis of FA-induced toxicological effects and the mechanism of silibinin improving cognitive impairment (Figure 11).

## Data Availability

The data used to support the findings of this study are included within the article.
